# Orthognathic Surgery after Mandibular Large-Volume Osteoma Treatment

**DOI:** 10.1155/2020/7310643

**Published:** 2020-01-13

**Authors:** Sabit Demircan, Sabri Cemil İşler, Aydın Gümüşdal, Begüm Genç

**Affiliations:** ^1^Private Practice, Beykent University Vocational School, Turkey; ^2^Istanbul University Faculty of Dentistry Oral and Maxillofacial Surgery Department, Turkey

## Abstract

Osteoma is a benign asymptomatic osseous tumor. Characterization of osteoma is the proliferation of cancellous or compact bone that increases in size by continuous formation of bone. It can be seen in any craniofacial structures, usually in paranasal sinuses and jaws. In this study, we present a 17-year-old male patient with a giant osteoma in the mandibular condyle. Posttreatment post-op follow-up, post-ortho, and orthognathic surgery after dental implantation are described.

## 1. Introduction

Osteoma is a benign osseous tumor arising from the proliferation of cancellous or compact bone that increases in size by continuous formation of bone. It is a slow-growing, asymptomatic, and usually solitary lesion which mainly affects young adults [1, 2].

Osteomas can be central, peripheral, or extraskeletal. Central osteomas arise from the endosteum, and a peripheral osteoma from the periosteum and extraskeletal soft-tissue osteomas usually develops within the muscle [2]. The cause of slow-growing osteomas is obscure, but the tumor may arise from cartilage or osseous periosteum; whether osteomas are benign neoplasm or hamartomas is not known [3]. Osteoma occurring in either the condyle or the condylar process may result in morphologic and functional disturbances, including facial asymmetry and temporomandibular joint dysfunction [4].

There are several treatment methods for osteomas. Usually, osteomas are asymptomatic, and leaving them alone is an option. Routine radiographic controls would be enough in these cases and osteomas might fade away. In some other cases, patients might have some symptoms or asymmetry, then nonsteroidal anti-inflammatory drugs (NSAIDs) support, curettage, radiofrequency ablation, or surgical treatment can be applied [5].

In this study, we present a 17-year-old male patient with a giant osteoma in the mandibular condyle. Posttreatment post-op follow-up, post-ortho, and orthognathic surgery after dental implantation are described.

## 2. Case Report

A systemically healthy male patient, 17 years old, with a prominent large swelling at the left mandibular ramus area leading to facial asymmetry which had developed over the previous 4 years, had referred to the Istanbul University, Faculty of Dentistry, Department of Oral and Maxillofacial Surgery. The swelling was not painful and no orofacial infection signs were reported. The patient had a history of head trauma due to falling off from a tree at the age of 6.

**Figure 1:**
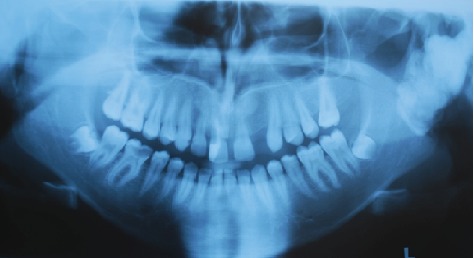
The initial panoramic radiograph of the patient.

**Figure 2:**
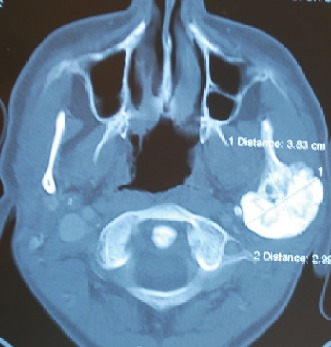
The initial CBCT of the patient.

**Figure 3:**
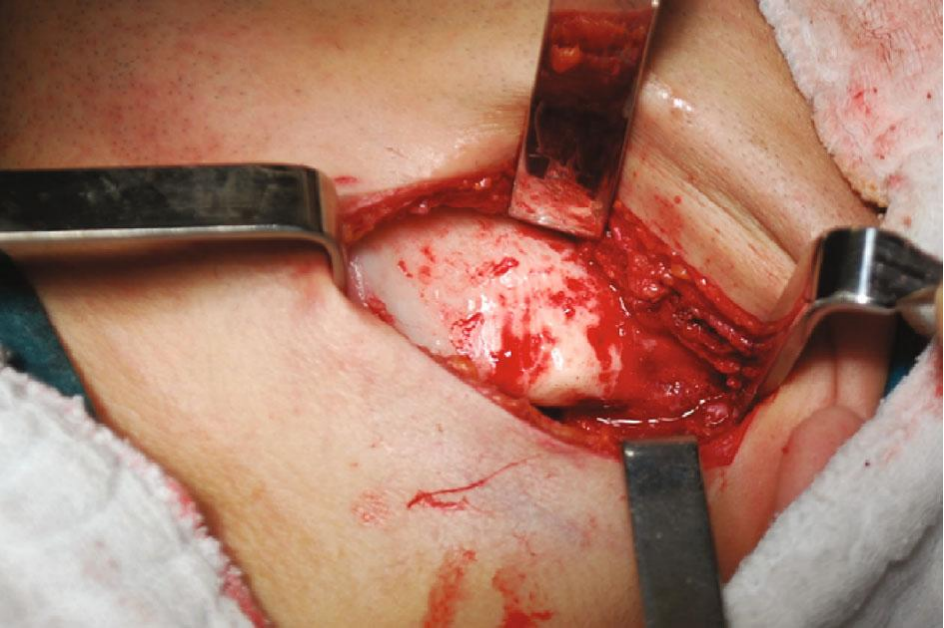
Extraoral approach to the lesion.

**Figure 4:**
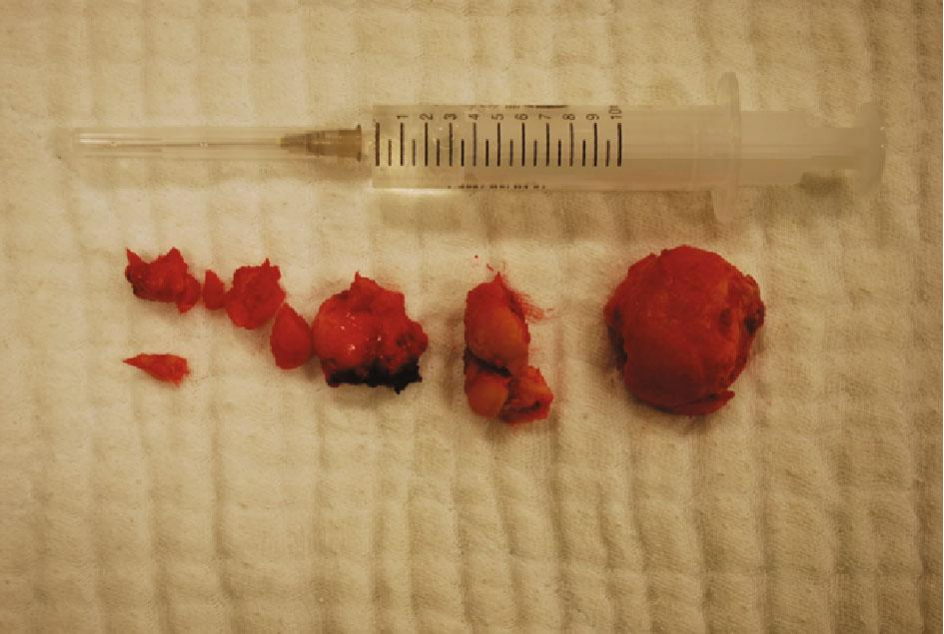
The removed lesions.

On extraoral examination, a hard, subcutaneous, multinodular, and painless mass of the complete left mandibular ramus was present. Deviation of the mandible to the right during mouth-opening and the protrusion are detected. During intraoral examination, there were no signs of pathology at the left mandible or the mandibular ramus area. With the radiological examination, the panoramic radiograph showed a large lobulated radiodense mass in the left mandibular ramus area and it also appeared to have a retromandibular partition (Figure 1). These findings were suggestive of a calcified odontogenic tumor affecting the jaw bone requiring a segmental or block resection. For further evaluation of the character and extent of the mass, Cone Beam Computed Tomography (CBCT) was performed. The CBCT scan showed a diffuse enlargement measuring 4.5 × 3.5 × 3 cm in the entire left mandibular ramus extending anteriorly to the mastoid process of the parietal bone (Figure 2). Because of the actual dysphagia, the facial asymmetry, and the progression of the lesion, it was decided to perform an operative correction to the left mandible. Under general anesthesia with nasoendotracheal intubation, the mandible was approached extraorally. 6 ml of 1/100000 adrenaline containing articaine HCl was injected to control the local hemorrhage. Using a fissure bur and a chisel, the lobular part of the lateral and medial side of the ascending ramus were resected and the mandible was reshaped (Figures 3 and 4). Besides this, healing was uneventful. Postoperative follow-up in the 6th month with dental volumetric tomography scans showed no evidence of pathology with an acceptable mandibular contour. In the next 5 years follow-up, there was no recurrence.

The patient also had class III skeletal malocclusion. After cephalometric analysis and malocclusion of the skeletal class III of the patient were detected, orthodontic treatment started. Following the initial treatment, the patient's wisdom teeth were extracted. The patient was made ready for orthognathic surgery.

7 years after the osteoma surgery, orthognathic surgery was performed. The bone at the left mandibular ramus area was fully regenerated and healthy. By performing two separate surgical operations instead of one operation, a possible bad split was avoided. Patient's age became proper for the surgery and the patient went through orthodontic treatment before the surgery. Two separate surgeries were comfortable for the patient. The only disadvantage of the separate surgeries was expensiveness of the treatment process.

**Figure 5:**
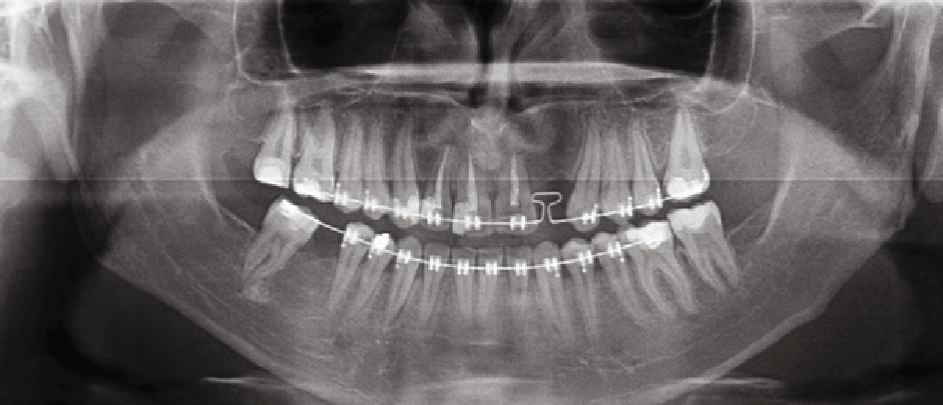
Panoramic radiograph of the patient before the orthognathic surgery.

**Figure 6:**
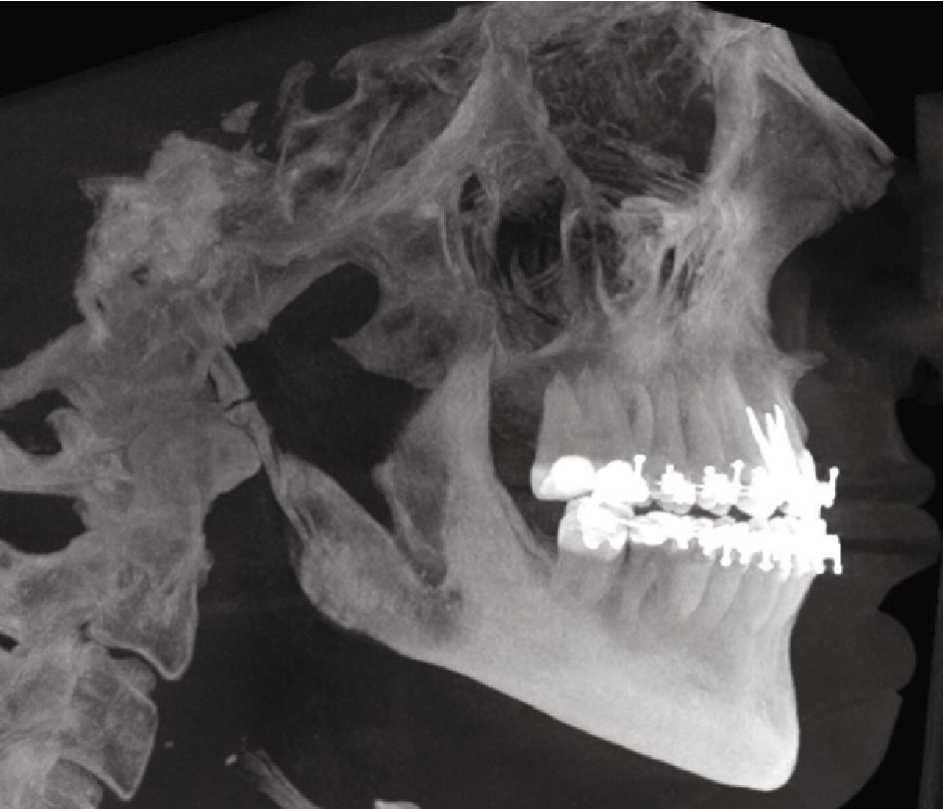
3D view of the patient before the orthognathic surgery.

**Figure 7:**
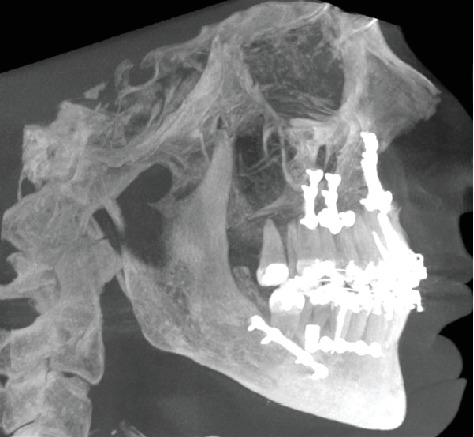
3D view of the patient after the orthognathic surgery.

**Figure 8:**
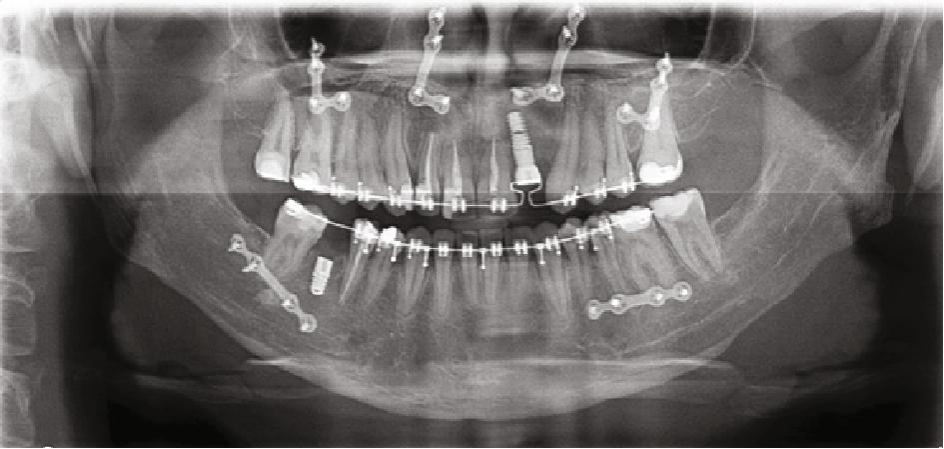
The panoramic radiograph of the patient after the implant surgery.

Maxilla Le Fort I osteotomy was performed with 3 mm anterior and 4 mm right, and mandibular bilateral sagittal split osteotomy was performed and 1 mm back and 2 mm left was taken (Figures 5–7). At the end of the first year following the operation, the patient's existing tooth deficiencies were rehabilitated by applying a dental implant (Figure 8).

## 3. Discussion

There is a very little understanding about the nature of osteomas, and three theories have been proposed: developmental, neoplastic, and reactive. It is unlikely that osteomas are a developmental anomaly, as most cases occur in adults and not during childhood or adolescence [3, 6]. It is also unlikely that osteomas are of a neoplastic nature, because of their very slow growth rate. The possibility of osteomas being a reactive lesion possibly resulting from local trauma is based on the history of trauma prior for the development of the lesion in some cases. However, this can be considered only in sites that are more susceptible to trauma, such as the angle or lower border of the mandible, but not in most of the cases. As many of the PO lesions are located in close proximity to muscle attachment (i.e., masseter, medial pterygoid, and temporalis), it is possible that muscle traction may play a role in the development of the PO [7]. The combination of trauma and muscle traction was also considered as a possible mechanism of the pathogenesis of osteomas [8, 9]. An accidental fall off a tree in the patient's history should have been considered as an etiology for this case.

Peripheral osteomas are uncommon. Clinically, the peripheral osteomas are usually asymptomatic slow-growing lesions which can produce swelling and asymmetry. The pathogenesis of peripheral osteomas is unclear [8, 10]. In the present case, the lesion was diagnosed at the age of 13. It has taken 4 years for the lesion to grow to this volume in accordance with the slow-growing characteristic.

Osteomas of the condyle may cause a slow progressive shift in occlusion with deviation of the midline of the chin towards the unaffected side and also lead to acute pain, limited mouth-opening, and malocclusion such as cross-bite [11]. Difficulties in swallowing and facial asymmetry were major complaints of the patient at the time of referral to our clinic, slow shifting of mandible to the right side was a symptom leading us to diagnose.

On radiological imaging, osteomas are well-defined radiopaque masses with distinct borders [12]. Osteomas composed solely of compact bone are uniformly opaque, while those containing cancellous bone show evidence of an internal trabecular structure [13]. They are smooth surfaced with a thin sclerotic rim. Imaging of osteomas can be achieved by traditional radiography (i.e., panoramic radiograph and Water's view) or by CT scan. The use of CT scanning with 3-D reconstruction makes it possible to achieve a better resolution and more precise localization [14, 15].

Removal of osteomas is not generally necessary. Surgery is indicated only when the lesion is symptomatic or actively growing [16, 17]. In our opinion, the surgical approach should be specific for cases. For the mandible, there are intraoral or extraoral approaches. The intraoral approach is preferable when possible, mainly for esthetic reasons [9].

During the initial few hours after general anesthesia, a temporary facial paralysis was observed due to the applied local anesthesia during the operation. This should not be misdiagnosed as a permanent facial paralysis [18].

In this case, in addition to a giant osteoma, there was also class III skeletal malocclusion. The osteoma should be removed because it caused asymmetry, and the malocculusion should be fixed by orthognathic surgery. The osteoma was very massive, and bone of the left mandibular ramus region was weak after osteoma removal. After waiting 7 years, the orthognathic surgery was performed. The separate surgeries were necessary to minimise risk of complications. With a separate surgery, bad split was avoided. The orthognathic surgery was comfortable for the patient and the surgeons. Expectedly, difficulty of elevation of the soft tissues was diagnosed. The bone of the left mandibular ramus region was totally regenerated and healthy. Performing a second surgery did not affect the degree of difficulty of the bilateral sagittal split osteotomy.

## 4. Conclusion

Osteomas are slowly growing pathological osseous structures; possible localizations are central, peripheral parts of bones or extraskeletaly, mostly in muscles. Osteomas in the mandibular region can cause problems such as deviation of mandible and limitation of masticator function. Treatment options vary depending on the degree of the asymmetry and functional disorders and the location and size of the lesion. Surgical approaches like blocking or total resection and corrective surgery of the lesions' contour should be considered when the “wait and see” option is inefficacious. Surgical approaches also have to be supported with histological findings. Recurrence or progress of the lesion should be examined with intermittent controls. If it is confirmed, the continuation of the dental treatment is recommended.
